# Streptococci and Scarlet Fever

**Published:** 1905-05-13

**Authors:** 


### STREPTOCOCCI AND SCARLET FEVER.

The true relation to scarlet fever of the streptococci so commonly present on the fauces of sufferers from this disease has long been, and still is, a vexed question. This much, however, appears certain, that whether or not these organisms can be held as the materies morbi of the disease itself, they undoubtedly play a large part in determining the common complications of the malady; and many people think the complications are more important than the disease. Dr. Alice Hamilton[1] has published the result of a research into the means by which streptococci may be disseminated by sufferers from scarlet fever, and if, as she in common with a good many observers believes, these organisms are responsible for the necrotic anginas, and the frequent lesions of the middle ear, nose, glands and kidneys, the matter is one of no little importance. Of the almost constant association of streptococci with scarlet fever there is no room for question. It is stated that in a series of 41 cases of the disease streptococci were found no less than 40 times; and that the infection not infrequently becomes septicæmic in fatal cases seems to be conclusively established by Baginsky and Sommerfeld who recovered this organism from the blood or internal organs of 82 fatal cases.

It is a commonplace of bacteriology that many non-virulent organisms may have their pathogenic properties exalted by passage through susceptible subjects, and thus it is highly probable that benign streptococci which are constant inhabitants of the healthy mouth may, under the influence of the inflammatory faucial changes associated with scarlet fever, become virulent to their host. Such organisms, again, by passage through other subjects, may have their virulence enhanced to the degree which provides the fulminating septic variety of scarlet fever, where the patient sinks in a day or two from acute intoxication. The bearing of this possibility on the correct treatment of scarlet fever in institutions where large numbers of patients are collected together needs little emphasis.

The method of investigation adopted in the study under notice consisted in the exposure, for definite intervals of time, of sterile media in Petri's capsules to the influence of the patient's expirations. Where possible, the subjects of the disease were asked to cough while the capsule was exposed a short distance in front of their mouths, and where, from age or illness, coughing was out of the question, the plate was merely submitted to the action of the breath. Positive results, in the shape of colonies of streptococci, were obtained in 66 per cent. of the experiments made. There is, of course, nothing new in the statement that all vigorous expiratory actions, whether breathing alone, or phonation in all its varieties, result in the ejection of invisible particles of saliva, which are competent to disseminate any organisms with which they may happen to be charged; such ejection is recognised as a fruitful source of calamity in the case of pulmonary tuberculosis, but the application of this knowledge to the prevention of complications in scarlet fever is somewhat insufficiently regarded. It is true that the authorities of our own fever hospitals consider that post-scarlatinal discharges from the nose and ears are infective, and act accordingly in postponing the dismissal of such patients, but Dr. Hamilton goes further in suggesting that all scarlet fever patients suffering from streptococcal complications should be segregated, and where for any reason such segregation is impracticable, she recommends the use of a gauze protector to prevent the ejection of infected particles from the air-passages.

[1] Jour. of American Med. Assoc., April 1905.

